# Advances in idiopathic pulmonary fibrosis diagnosis and treatment

**DOI:** 10.1016/j.pccm.2025.02.001

**Published:** 2025-03-07

**Authors:** Hongli Liu, Jiaxi Shen, Chao He

**Affiliations:** a Division of Pulmonary, Allergy, and Critical Care Medicine, Department of Medicine, University of Alabama at Birmingham, Birmingham, AL 35294, USA; b Section of Pulmonary, Critical Care, and Sleep Medicine, Department of Medicine, Baylor College of Medicine, Houston, TX 77024, USA

**Keywords:** Idiopathic pulmonary fibrosis, Biomarkers, Cryobiopsy, Genomic classifier, Clinical trials

## Abstract

Significant advances have been made in diagnosing and treating idiopathic pulmonary fibrosis (IPF) in the last decade. The incidence and prevalence of IPF are increasing, and morbidity and mortality remain high despite the two Food and Drug Administration (FDA)-approved medications, pirfenidone and nintedanib. Hence, there is an urgent need to develop new diagnostic tools and effective therapeutics to improve early, accurate diagnosis of IPF and halt or reverse the progression of fibrosis with a better safety profile. New diagnostic tools such as transbronchial cryobiopsy and genomic classifier require less tissue and generally have good safety profiles, and they have been increasingly utilized in clinical practice. Advances in artificial intelligence-aided diagnostic software are promising, but challenges remain. Both pirfenidone and nintedanib focus on growth factor-activated pathways to inhibit fibroblast activation. Novel therapies targeting different pathways and cell types (immune and epithelial cells) are being investigated. Biomarker-based personalized medicine approaches are also in clinical trials. This review aims to summarize recent diagnostic and therapeutic development in IPF.

## Introduction

Idiopathic pulmonary fibrosis (IPF) is a chronic and progressive fibrosing interstitial lung disease (ILD) with high mortality and an average life expectancy of 3–5 years after initial diagnosis.^1^ Additionally, there is an increasing incidence and prevalence of IPF and other ILDs.^2^ Historically viewed as a disease caused by chronic inflammation, the current consensus is that IPF is initiated by aberrant epithelial cell injury/expansion and dysregulated repair, which lead to the recruitment and activation of profibrotic monocyte-derived alveolar macrophages (MoAMs) and fibroblasts.^3^ This has shifted therapies from immunosuppressive treatments to anti-fibrotic drugs like pirfenidone and nintedanib, which slow but do not halt disease progression. While they reduce disease progression, neither stops nor reverses the decline in lung function. Hence, there remains an urgent need to develop new diagnostic tools and effective therapeutics to improve early, accurate diagnosis of IPF, and halt or reverse the progression of fibrosis with a better safety profile. Recent genetic and non-genetic risk factors offer a foundation for personalized medicine, and repurposing drugs that target fibroproliferative pathways presents further therapeutic potential.

## Diagnostic advances for IPF

The high mortality of IPF underscores the heightened necessity of early and accurate diagnostic processes. High-resolution computed tomography (HRCT) plays a pivotal role in the initial assessment of individuals suspected of having IPF and significantly impacts the subsequent diagnostic process. Surgical lung biopsy (SLB) has remained the mainstay and gold standard of IPF diagnosis for decades. However, with recent advances in HRCT, in patients who exhibit either a usual interstitial pneumonia (UIP) pattern or a probable UIP pattern on HRCT scans without any characteristics that would rule out a diagnosis of IPF, a diagnosis of IPF can be established following a comprehensive multidisciplinary discussion (MDD), eliminating the need for surgical lung biopsy.^4^ This is recommended in the most recent 2022 American Thoracic Society (ATS)/European Respiratory Society (ERS)/Japanese Respiratory Society (JRS)/Asociación Latinoamericana de Tórax (ALAT) guidelines for IPF and progressive pulmonary fibrosis (PPF) diagnosis in adults.^5^ However, a definite UIP pattern is only present in one-third of the patients.^6^ A substantial proportion of patients with IPF do not present with typical HRCT features of a UIP pattern, and diagnosis could be challenging. Hence, for these patients presenting with indeterminate UIP patterns, tissue sampling is still required for definitive diagnosis, given the poor outcome of IPF. *Post hoc* subgroup analysis from the INPULSIS trial showed that there was no difference in the annual rate of decline in forced vital capacity (FVC) or treatment response to nintedanib in those with IPF diagnosis without UIP pattern on HRCT, compared to those with honeycombing on HRCT and/or confirmation of UIP by biopsy.^7^

In recent years, substantial advances have facilitated the development of novel diagnostic tools for patients with IPF, incorporating new lung sampling methods, pathologic and biologic features, and improved imaging techniques and interpretation.^8^

### Transbronchial lung cryobiopsy (TBLC)

Histologically, the UIP pattern is characterized by patchy dense fibrosis with remodeling of lung architecture, resulting in microscopic honeycomb with temporal heterogeneity. Due to its propensity for selectively sampling subpleural regions and yielding larger tissue specimens, SLB is traditionally favored as the method of choice for tissue sampling.^4^ Traditional transbronchial lung biopsy samples are small and subject to significant crush artifacts, preventing a detailed study of tissue architecture and limiting its diagnostic value for IPF.^9^ TBLC yields larger biopsy specimens (43 to 64 mm^2^) compared with transbronchial biopsy (5.8 mm^2^) without crush artifacts, and therefore is more likely to yield diagnostic information.^10^, ^11^, ^12^ Studies have shown that TBLC improves diagnostic confidence and histopathologic diagnostic yield. A cross-sectional study involving 117 patients with fibrotic ILDs without a UIP pattern on HRCT examined the impact of TBLC on diagnostic confidence in MDD. Compared to clinical information alone, the study found that diagnostic confidence increased from 29 % to 63 % in the TBLC group.^13^ An additional study observed a comparable increase in diagnostic confidence when TBLC was incorporated alongside bronchoalveolar lavage (BAL) in the diagnostic process.^14^ A comprehensive meta-analysis encompassing 39 studies investigated the diagnostic efficacy of TBLC in ILD patients.^15^ Overall, TBLC achieved a diagnostic yield of 80 % (with a 95 % confidence interval of 76 % to 84 %), although there was notable inconsistency across different studies. In conclusion, the diagnostic yield of TBLC is comparable to the diagnostic yield of SLB, which is close to 90 % based on previously published studies.^4^ Three prospective studies have examined the diagnostic concordance between TBLC and SLB.^16^, ^17^, ^18^ In these studies, patients with ILD of uncertain type who were referred for SLB received paired TBLC and SLB. The agreement between the diagnostic interpretations of the TBLC and SLB sample was then assessed. The largest of the three studies, the COLDICE study,^18^ which enrolled 65 patients, reported a high level of agreement between TBLC and SLB for both histopathological interpretation (70.8 %) and multidisciplinary discussion (76.9 %). The concordance increases if (1) TBLC MDD diagnoses were made with high confidence, (2) a UIP pattern was identified on SLB, or (3) an increased number of samples were obtained from TBLC.^19^ SLB could only make a confident diagnosis in 6 out of the 40 cases (23 %) where the results of TBLC were non-diagnostic.^18^ The other two smaller studies reported a moderate agreement (61.7 %) and a poor agreement (48 %), respectively.^16^^,^^17^ Of the three studies, COLDICE enrolls the largest number of patients and uses consensus pathology reports and centralized MDD, offering better generalizability compared to the other two studies.^18^ However, significant methodological variances among the three studies, including trial design and statistical analysis, render direct comparisons of their results potentially difficult. The characteristics of the study design and results of these three studies are summarized in Table 1. Common complications of TBLC include pneumothorax (7 %) and any bleeding (39 %).^15^ Severe bleeding, respiratory failure, respiratory infection, persistent air leak, and mortality are rare. The safety of TBLC was also studied in patients deemed at higher risk for the biopsy procedure, defined as body-mass index >35 kg/m^2^, age >70 years, severely impaired lung function (FVC < 50 % pred or diffusing capacity for carbon monoxide [DLCO] <30 % pred), systolic pulmonary artery pressure >45 mmHg or clinically significant heart disease. The results showed no significant difference in complication rates, such as bleeding or pneumothorax, between high-risk and low-risk groups.^19^^,^^20^

**Table 1:** Comparison of studies investigating transbronchial lung cryobiopsy (TBLC).

Items	COLDICE^18^	CANICE^16^	Cryo-IPF^17^
Patient number	65	20	21
Multicenter	Yes (9 centers)	Yes (3 centers)	Yes (2 centers)
Pre-procedural MDD	Centralized	Per center	Per center
Pathology review	Consensus from 3 pathologists	60 paired results from 3 pathologists’ independent review	Single pathologist
Pathology report	Specific ILD histopathologic pattern and guideline-directed histopathologic pattern	Specific ILD histopathologic pattern	Specific ILD histopathologic pattern
Post-procedural MDD	Centralized MDD	Independent review at 3 centers	Per center, incorporating both TBLC and SLB
Primary outcome	Co-primary endpoints: (1) agreement of histopathological interpretation using guideline-directed histopathologic pattern; (2) diagnostic agreement between TBLC-MDD and SLB-MDD	Diagnostic agreement between TBLC-MDD and SLB-MDD	Diagnostic agreement between TBLC and SLB for specific ILD diagnosis
Results			
Percent of patients with diagnosis of IPF at SLB-MDD	53.8 %	26.7 %	42.9 %
Diagnostic agreement between TBLC-MDD and SLB-MDD	Incidence 76.9 %, Cohen's κ 0.62 (95 % CI 0.47 – 0.78)	Incidence 61.7 %, Cohen's κ 0.38 (95 % CI 0.29 – 0.63)	Not applicable
Histopathologic agreement between TBLC and SLB for specific ILD diagnosis	9 different pathologic categories Incidence 69.2 %, Cohen's κ 0.47 (95 % CI 0.30 – 0.64)	4 different pathologic categories Incidence 56.7 %, Cohen's κ 0.38 (95 % CI 0.22 – 0.53)	11 different pathologic categories Incidence 38 %, Cohen's κ 0.22 (95 % CI 0.01 – 0.44)
Histopathologic agreement between TBLC and SLB for guideline-directed histopathologic pattern	Incidence 70.8 %, weighted Cohen's κ 0.70 (95 % CI 0.55 – 0.86)	Not applicable	Not applicable

CI: Confidence interval; ILD: Interstitial lung disease; IPF: Idiopathic pulmonary fibrosis; MDD: Multidisciplinary discussion; SLB: Surgical lung biopsy.

In summary, TBLC is a valuable tool in IPF diagnosis. It exhibits a comparable diagnostic yield to SLB and increases diagnostic confidence at MDD with relatively low complication rates. Moreover, the procedure may be safely performed in those patients in whom SLB may be deemed high risk due to significant comorbidities. The updated 2022 ATS/ERS/JRS/ALAT guideline recommended that TBLC can be considered as an acceptable alternative to SLB to obtain histopathological samples in patients with ILD of undetermined type in selected medical centers with experience in performing and interpreting TBLC (conditional recommendation, very low quality evidence).^5^ In a separate 2022 ERS guideline, there is a stronger endorsement for the use of TBLC.^21^ This guideline suggests that TBLC should be the preferred method for patients with undiagnosed ILD considered eligible to undergo SLB (conditional recommendation, very low certainty of evidence).

### Genomic classifier

The genomic classifier is a tool used in precision medicine that analyzes genetic data to provide insights into diseases or conditions. It typically involves sequencing and analyzing DNA or RNA from patient samples to identify specific genetic patterns, mutations, or expression profiles. The Envisia Genomic Classifier analyzes genetic material from lung tissue samples obtained through less invasive transbronchial biopsies. The core technology involves Next Generation RNA Sequencing to evaluate RNA extracted from these biopsy samples. The test examines gene expression data from 190 genes and employs a machine learning algorithm to distinguish two genetic subtypes: usual interstitial pneumonia (UIP) and non-UIP.^22^

The Envisia Genomic Classifier was examined in two separate multicenter validation studies derived from the Bronchial Sample Collection for a Novel Genomic Test (BRAVE) cohort.^23^^,^^24^ In both studies, the patients were divided into training and validation cohorts. Taken together, the sensitivity and specificity of identifying UIP on a transbronchial lung sample was 62.5 % and 90.6 %.^25^

The clinical utility of the Envisia Classifier was primarily assessed in patients suspicious of IPF but lacking a UIP pattern on HRCT. In the CATALYST study, 220 patients were recruited from the BRAVE cohort. At the same time, 124 were excluded from the final analysis due to the absence of diagnostic molecular test results or diagnostic pathology.^24^ Twenty-four patients who did not exhibit a typical UIP pattern on HRCT tested positive for UIP using the Envisia Classifier. Thus, incorporating the genomic classifier results increased the sensitivity of identifying a histopathologic UIP pattern from 34 % to 79.2 % in patients without UIP pattern on HRCT. The second study included 11 individuals with histopathologic UIP, and positive genomic classifier results for UIP, but without typical UIP manifestations on HRCT.^26^ The genomic classifier increased diagnostic sensitivity from 30 % to 69 % and led to a higher chance of recommending anti-fibrotic treatment (46.4 % compared to 10 %). Another study, which included 24 patients without a definitive UIP pattern on HRCT, demonstrated that adding Envisia Classifier results to TBLC markedly improved the diagnostic confidence for IPF from 43 % to 93 % at MDD.^27^

In summary, the Genomic Classifier demonstrates high specificity for the histopathologic UIP pattern, though its sensitivity remains moderate. When applied to a population with moderate to high pretest probability of IPF, the high specificity of the Genomic Classifier indicates a strong positive predictive value (PPV), suggesting that a positive result is highly reliable in confirming the presence of the histopathologic UIP pattern. However, due to its moderate sensitivity, the negative predictive value (NPV) is comparatively lower, implying that a negative result may not conclusively rule out UIP. Therefore, while positive results can be interpreted with confidence, negative findings should prompt further diagnostic evaluation in cases where clinical suspicion of UIP remains high. Also, the Genomic Classifier is unable to differentiate fibrotic ILD subtypes. Together with TBLC, they improve the diagnostic confidence in IPF during MDD. Additionally, it may hold significance for patients who lack a characteristic UIP pattern on HRCT and have nondiagnostic biopsy results. Nevertheless, it is important to note that existing studies on this subject are limited in scale. Moreover, they uniformly employ pathology as the definitive reference, thereby not fully exploring the additional diagnostic value that the genomic classifier might offer beyond conventional clinical and pathological investigations. The 2022 ATS/ERS/JRS/ALAT guidelines made no recommendation for or against the addition of genomic classifier testing for the purpose of diagnosing UIP in patients with ILD of undetermined type who are undergoing transbronchial forceps biopsy, because of insufficient agreement among the committee members.^5^

### Artificial intelligence (AI) technologies

Advances in AI, particularly in radiology, have significantly accelerated AI-assisted diagnosis in various disease conditions. Studies have shown that the application of artificial intelligence, mostly using computer-based deep learning technologies, has the potential to supplement or even enhance the accuracy of human-led diagnoses in ILDs.

***Omics-based analysis:*** A meta-analysis including 19 retrospective studies examined the accuracy of AI classifying ILDs into different radiographic patterns. The accuracies reported ranged from 78 % to 91 %, with two studies demonstrating near-expert performance in diagnosing IPF.^28^ Another study involved a retrospective analysis of 1,239 patients with biopsy-proven ILD and chest CT imaging. A deep learning model was developed and trained to predict UIP or non-UIP histopathology based on CT images. Compared with two subspecialty-trained radiologists, the model demonstrated superior prediction of histopathologic UIP and excellent reproducibility.^29^ The accuracy and reliability of AI technology to identify pulmonary fibrosis with a rapid per-case processing time has led to the development of the software ScreenDx-LungFibrosis™, which has the potential to be used routinely in CT analysis.^30^ AI-assistance image analysis can also help to predict disease progression and mortality.^31^ The study showed several CT features (lower lung volume, increased vascular volume, and increased fibrosis volume) are associated with progression-free survival in the UK-based PROFILE cohort. In addition to radiomics, machine learning has been used in plasma proteomics to differentiate IPF and non-IPF ILD.^32^

***Electronic health record (EHR)-based analysis:*** Another study developed a new screening tool called the ZCoR-IPF. It utilizes EHR to identify subtle comorbidity incidence, timing, and sequence characteristics preceding IPF. The tool was trained on a national insurance claims database and validated with three independent databases, involving over 2.98 million participants, including 54,247 positive IPF cases. The main advantage of EHR-based analysis is that it is noninvasive and inexpensive. Using existing data, the algorithm can predict a diagnosis of IPF with robust confidence (positive likelihood ratio >30 at a specificity of 0.99) for both sexes with different comorbidities up to 4 years in the future. The authors concluded that if adopted, ZCor-IPF can allow earlier diagnosis in at-risk populations and improved outcomes of interventions.^33^

Despite the significant promise of AI in the diagnosis of IPF, challenges remain before these technologies can be routinely integrated into clinical practice. One of the primary obstacles is the quality and availability of data required to train AI models effectively. Given the rarity of IPF, assembling large, high-quality datasets that reflect diverse patient populations can be difficult. Additionally, ensuring that AI models are trained on datasets that are representative of different ethnicities, comorbidities, and disease severities is essential for developing models that generalize well across various clinical settings.

Another challenge lies in integrating AI into real-time clinical workflows. While AI systems have demonstrated impressive accuracy in controlled, retrospective analyses, implementing these systems in the fast-paced environment of clinical care requires overcoming logistical hurdles, such as interoperability with existing electronic health record systems, ensuring timely data processing, and providing clinicians with actionable insights that are easily interpretable. Moreover, economic factors like the cost-effectiveness of deploying AI technologies in routine practice, especially in resource-limited settings, must be considered. While AI holds the promise of reducing diagnostic delays and improving outcomes, its widespread adoption may be hindered by initial costs and the need for infrastructure updates.

Finally, regulatory and ethical considerations present additional layers of complexity. Regulatory bodies must establish clear frameworks for evaluating the safety and efficacy of AI in healthcare, ensuring that AI systems meet rigorous standards before being deployed in clinical environments. Ethical concerns surrounding the use of AI in healthcare, particularly in terms of patient privacy, data security, and the potential for bias in AI algorithms, must also be addressed. Ensuring that AI systems are transparent, explainable, and free from biases is critical for maintaining trust between patients and healthcare providers.

To summarize, artificial intelligence offers transformative potential in the diagnosis of IPF, with advancements in image-based and EHR-based analyses leading the way. AI-driven diagnostic tools such as deep learning models for CT imaging and EHR-based algorithms like ZCoR-IPF are showing promising results in improving diagnostic accuracy, reducing delays, and identifying high-risk patients earlier. However, for AI to become a routine part of IPF management, significant challenges related to data quality, clinical integration, cost, and regulatory approval must be addressed. Overcoming these obstacles will be key to fully harnessing AI's potential to improve outcomes for patients with IPF and other ILDs.

### Endobronchial optic coherence tomography (EB-OCT)

Optical coherence tomography (OCT) utilizes low-coherence light waves as a non-ionizing imaging method, enabling high-resolution images of soft tissues with a resolution of <10 micrometers. EB-OCT involves inserting an OCT probe *via* the working channel of a flexible bronchoscope, allowing for the *in vivo* examination of peripheral and subpleural lung tissues.^34^^,^^35^ Historically, the technology was used in patients with asthma and other airway diseases to assess airway caliber, extent of airway remodeling and quantify airway mucus.^35^ There are emerging data suggesting that EB-OCT could be useful in IPF diagnosis.

In a pilot study of 5 patients undergoing surgical lung biopsy, EB-OCT can detect and distinguish microscopic features of UIP/IPF and non-UIP/IPF ILD, including fibrosis extent and distribution, microscopic honeycombing (<2 mm) not visualized by HRCT, and traction bronchiectasis confused with honeycombing on HRCT.^36^ The diagnostic accuracy of EB-OCT for IPF was further investigated in a study enrolling 27 patients undergoing surgical lung biopsy (12 were eventually diagnosed with UIP). EB-OCT was performed immediately before the lung biopsy and was blinded to the researchers. The sensitivity and specificity of EB-OCT was 100 % (95 % confidence interval [CI] 75.8 %–100 %) and 100 % (95 % CI 79.6 %–100 %) respectively, for both histologic UIP and clinical diagnosis of IPF.^37^

Research has been conducted to assess the efficacy of polarization-sensitive (PS)-EB-OCT, an advanced derivative of conventional OCT.^35^^,^^38^ This enhancement introduces tissue-specific contrast by evaluating birefringence presence. Birefringence, an inherent optical characteristic displayed by collagen within various tissues, is notably prominent in fibrotic lung conditions.^39^ Leveraging biopsy findings as a comparative benchmark, PS-EB-OCT demonstrated its capacity to accurately predict fibrosis levels observed in biopsy specimens and effectively discriminate between IPF and non-IPF ILDs.^38^ However, larger multicenter studies are needed before EB-OCT can be implemented in routine clinical practice. Further consideration is needed for technical challenges, such as establishing validated methods to confirm the accurate positioning of the subpleural EB-OCT probe.^40^ Although EB-OCT holds promise for diagnosing IPF, its broader application faces hurdles including the need for specialized training, limitations in image resolution, technological and procedural challenges, and the absence of extensive clinical validation. These obstacles highlight the critical need for more research, educational initiatives, and well-defined strategies for its integration. Advancing these areas is essential for leveraging EB-OCT's full potential in diagnosing IPF.

### Biomarkers for differential diagnosis of IPF

Thus far, several biomarkers have demonstrated differential expression in patients with IPF compared to healthy controls. These biomarkers encompass dysfunction of alveolar epithelial cells, remodeling of the extracellular matrix, dysregulation of the immune system, and cascades of inflammation.

Some of these biomarkers also exhibit differential expression in IPF compared to non-IPF ILDs. For instance, in a study that examined the gene expression profile of explanted lungs from biopsy-proven cases of clinical diagnosis of IPF and non-specific interstitial pneumonia (NSIP), insulin-like growth factor binding protein (IGFBP)-5 and 6, Mucin 5B, and actin alpha 2 (ACTA-2) were specific for IPF.^41^ In another study, plasma surfactant protein (SP)-D, matrix metalloproteinase (MMP)-7, and osteopontin each significantly distinguished patients with IPF from patients with alternative ILD (which mainly included NSIP and uncharacterized fibrosis), both individually and in a combined index.^42^ Serum concentrations of MMP-28 were significantly higher in IPF versus non-IPF fibrosis (mostly connective tissue disease [CTD]-ILD and chronic hypersensitivity pneumonitis).^43^ As reviewed by Wang et al,^44^ >100 biomarkers have been identified across various biological samples, including serum, BAL fluid, lung tissue, and sputum, in studies of IPF published between 2005 and 2020. Below are some of the most extensively studied biomarkers with potential to distinguish IPF from other chronic interstitial lung diseases, summarized in Table 2. However, this represents only a subset of the available literature and is not an exhaustive compilation of the published data.

**Table 2:** Selected biomarkers for IPF.

Biomarker	Cellular function	Clinical significance
Club cell protein 16 (CC16)	Anti-inflammatory and antioxidative effects	Upregulated in both serum and bronchoalveolar lavage of IPF patients compared to non-IPF ILDs^45^, ^46^, ^47^, ^48^
Matrix metalloproteinases (MMPs)	Extracellular matrix remodeling	MMP-1, MMP-7, and MMP-28 exhibit greater specificity for IPF versus other ILDs^42^, ^43^, ^44^^,^^50^^,^^51^
Mucin 5B (MUC5B)	Mucin production by airway secretory cells	The rs35705950 SNP is associated with increased risk for IPF and familial pulmonary fibrosis (FPF), not associated with CTD-ILD^54^, ^55^, ^56^, ^57^, ^58^, ^59^, ^60^, ^61^

CTD: Connective tissue disease; ILD: Interstitial lung disease; IPF: Idiopathic pulmonary fibrosis; SNP: Single nucleotide polymorphism.

***Club cell protein 16 (CC16):*** CC16 is a 16 kDa homodimeric protein secreted primarily by club cells, the non-ciliated bronchiolar secretory cells in the respiratory epithelium.^45^ Studies have shown that CC16 exhibits anti-inflammatory and antioxidative effects in different types of cells. CC16 was upregulated in both serum and BAL fluid from IPF subjects.^46^ Additionally, it has been identified as a marker of pulmonary fibrosis in patients with combined pulmonary fibrosis and emphysema (CPFE) and systemic sclerosis-associated pulmonary fibrosis.^47^^,^^48^

***Matrix metalloproteinases (MMPs):*** MMPs are calcium-dependent zinc-containing endopeptidases extensively involved in extracellular matrix remodeling. In individuals with IPF, several members of the MMP family, including MMP-1, MMP-3, MMP-7, MMP-9, and MMP-28, have been implicated compared to healthy controls.^49^ Some research indicates that MMP-1, MMP-7, and MMP-28 may exhibit greater specificity to IPF compared to other non-IPF ILDs.^42^^,^^43^^,^^50^ However, contradictory results exist. For instance, in a comparison between rheumatoid arthritis-associated ILD (RA-ILD) and IPF, MMP-7 levels were found to overlap, while MMP-1 was observed to be more prevalent in RA-ILD patients.^51^ MMP-7 was recently identified as a plasma marker for epithelial cell injury in IPF and is associated with the greatest FVC decline.^52^

***Mucin 5B** (MUC5B): MUC5B* is responsible for encoding a precursor protein of mucin 5B, a key gel-forming mucin secreted by proximal submucosal glands and distal airway secretory cells.^53^ Single nucleotide polymorphism (SNP) located in the *MUC5B* promoter on chromosome 11, rs35705950, was linked to elevated *MUC5B* expression.^54^ Importantly, this SNP was also associated with developing IPF and familial pulmonary fibrosis (FPF). Subsequent research, including numerous cohorts and genome-wide association studies (GWAS), has consistently confirmed these findings.^55^, ^56^, ^57^ Additionally, this polymorphism does not demonstrate an association with lung fibrosis in systemic sclerosis,^58^ sarcoidosis,^59^ or autoimmune myositis.^60^ However, it has been linked to fibrotic hypersensitivity pneumonitis.^61^

Although biomarkers show significant promise, they have not become a standard component in the clinical differential diagnosis of IPF. Existing studies are predominantly retrospective, with limited patient enrollment and a lack of a universally accepted gold standard for diagnosing other ILDs. The relative specificity of each biomarker for IPF is also likely affected by ethnicity, as indicated by findings from a global multi-ancestry meta-analysis.^62^

In summary, current diagnostic advances in IPF have improved precision, but challenges remain. While HRCT and MDD have reduced the need for surgical lung biopsies in patients with a probable or definite UIP pattern, a significant proportion of IPF patients present with atypical patterns, necessitating further tissue sampling. Techniques like transbronchial lung cryobiopsy have emerged as a valuable alternative to surgical biopsies, offering comparable diagnostic yields with fewer complications. However, the variability in TBLC diagnostic agreement with surgical biopsies across studies and procedural risks highlight the need for refinement of these methods.

Additionally, tools like the Envisia Genomic Classifier, which uses RNA sequencing to identify UIP patterns, offer promise but are limited by moderate sensitivity, necessitating additional research to optimize its utility in diagnostic workflows. Similarly, while artificial intelligence (AI)-assisted tools have shown great potential in improving diagnostic accuracy through image analysis and electronic health records, there are significant hurdles in integrating AI into real-time clinical practice, including data quality, cost, and regulatory concerns.

Future directions in the diagnosis of IPF should focus on further improving non-invasive diagnostic tools, expanding the utility of biomarkers, and integrating AI technologies in a way that is feasible and accessible across diverse clinical settings. Additionally, larger, multicenter studies are needed to validate novel technologies like EB-OCT and genomic classifiers, ensuring that they can be adopted into routine clinical practice. Addressing these challenges will be critical to achieving earlier and more accurate diagnoses, ultimately improving patient outcomes.

## Advances of drug therapy for IPF

IPF has long been considered a chronic inflammatory condition. However, the National Institute of Health (NIH)-sponsored double-blinded, randomized, placebo-controlled PANTHER trial, which evaluated the benefits of the combined triple therapy of prednisone, N-acetylcysteine (NAC), and azathioprine (Imuran), was terminated earlier due to increasing mortality and rate of acute exacerbation in the experimental group.^63^ The failure of the PANTHER trial marked a significant shift in developing therapies for IPF. The current concept is that IPF is a complex disease that begins with repeated epithelial injuries that lead to the recruitment of pro-fibrotic macrophages and activation of (myo)fibroblasts. Hence, there is significant interest in developing anti-fibrotic, rather than anti-inflammatory, therapeutics targeting growth factors such as transforming growth factor-β (TGF-β).

Two novel anti-fibrotics, namely pirfenidone and nintedanib, have both demonstrated a significantly reduced decline in lung function compared to placebo.^64^, ^65^, ^66^ Nintedanib, a tyrosine kinase inhibitor, targets multiple growth factor signaling pathways.^67^ The exact target of pirfenidone remains unclear.^68^ Food and Drug Administration (FDA) approved both medications for treating IPF in 2014, and both medicines received conditional recommendations from ATS/ERS practice guidelines in 2015.^69^ While pirfenidone and nintedanib reduce disease progression, they do not stop or reverse the decline in lung function. Over the past decade, several trials have examined novel compounds as an alternative, replacement, or add-on therapy for IPF (Table 3).

**Table 3:** Drug development for IPF.

Drugs	Mechanism(s)	Results	Status
Compounds currently studied in phase 3 stage			
αvβ6 integrin inhibitors (BG00011, PLN-74809)	Inhibition of TGF-β activation^72^^,^^73^	BG00011 showed a worsening trend in IPF^78^^,^^79^ PLN-74809 showed a reduction in FVC decline and less fibrosis progression^80^	Phase 2b trial of BG00011 was terminated early; Phase 3 trial ongoing for PLN-74809 (BEACON-IPF)
BI 1015550	Phosphodiesterase type 4 (PDE4) inhibitor^83^	Slow FVC decline with or without antifibrotic treatment^82^, ^83^, ^84^	Phase 3 met its primary endpoint of slowing FVC decline at 26 weeks^84^ (FIBRONEER IPF)
LPA1 antagonist	Inhibit fibroblast recruitment^85^	BMS 986020 showed a reduction in FVC decline but adverse events led to early termination; BMS 986278 was well-tolerated^86^^,^^87^	Phase 3 trial ongoing for BMS 986278, and expected to be completed in 2026^87^
Phosphodiesterase 5 inhibitor	Inhaled prostacyclin analog	Showed improvement in 6-min walk test and FVC in PH-ILD patients^88^^,^^89^	Phase 3 trial ongoing (TETON, TETON-2)
N-acetylcysteine (NAC)	Antioxidant^90^^,^^91^	Mixed results in preserving FVC in IPF population May have benefits in patients with certain genetic background^90^, ^91^, ^92^, ^93^, ^94^, ^95^, ^96^, ^97^, ^98^	Phase 3 trial targeting patients with specific genetic mutations (*TOLLIP* TT genotype) (PRECISIONS trial)
Compounds failed in phase 2b/3 stage			
Ziritaxestat (GLPG1690)	Autotaxin inhibitor	Improvement in FVC^100^	Phase 3 trials (ISABELA 1 and 2) terminated early due to futility. Higher all-cause mortality rates compared to placebo
Pamrevlumab (FG-3019)	Recombinant antibody to connective tissue growth factor	Decreased decline in FVC by approximately 70 % at 48 weeks in phase 2 trial^101^	Phase 3 trial (ZEPHYRUS-1) failed to meet primary endpoint (mean decline in FVC)^102^

FVC: Forced vital capacity; IPF: Idiopathic pulmonary fibrosis; LPA1: Lysophosphatidic acid receptor-1; PH-ILD: Pulmonary hypertension associated with interstitial lung disease; TGF-β: Transforming growth factor-β; *TOLLIP*: Toll-interacting protein.

### Combination of pirfenidone and nintedanib

There has been growing interest in combining pirfenidone and nintedanib to treat IPF. Safety and tolerability studies investigating the combination therapy of pirfenidone and nintedanib have been promising, where combination therapy was similarly tolerated with a manageable safety profile compared to monotherapy.^70^^,^^71^ The open-labeled randomized INJOURNEY trial compared the safety and efficacy of monotherapy nintedanib (150 mg twice daily) *vs.* combination nintedanib (150 mg twice daily) and add-on pirfenidone (titrated to 801 mg three times daily) over 12 weeks in IPF patients. The primary endpoint is the treatment-related gastrointestinal adverse events, and the efficacy endpoints are exploratory. There was no increase in adverse events in combination therapy compared to monotherapy, on top of an observation of about a 50 % reduction in the rate of decline in lung function (mean change in FVC: −13.3 mL in combination therapy *vs.* −40.9 mL nintedanib alone). Reported adverse events (any) were 88.7 % in the combination treatment group and 88.2 % in the monotherapy group.^70^ A similar safety profile is reported when nintedanib is used as an add-on therapy for patients who are on stable doses of pirfenidone.^71^ Neither study was powered to evaluate the efficacy of combination therapy, and no control groups were included. There is no recommendation from society guideline regarding combination therapy.

### Compounds currently studied in phase 3 stage

***Integrin inhibitors:*** Integrins are heterodimer transmembrane proteins. αv integrins activate latent TGF-β *via* binding to the arginyl-glycyl-aspartic acid (RGD) sequence in the latency-associated peptide (LAP) of TGF-β.^72^^,^^73^ Targeting αv integrin genetically or pharmaceutically attenuated pulmonary fibrosis.^74^ αvβ6 expression is increased in alveolar epithelial cells from IPF patients, compared with healthy subjects.^75^ αvβ6 neutralizing antibodies protect mice from developing pulmonary fibrosis after bleomycin exposure or radiation as well as in TGF-β overexpression transgenic mice.^75^, ^76^, ^77^ Therapeutically targeting αvβ6 has been examined in several clinical trials. A trial using a subcutaneous injection of humanized anti-αvβ6 IgG1 monoclonal antibody (BG00011) was terminated in 2019 after a phase 2 trial. IPF subjects receiving BG00011 showed a worsening trend of FVC after 26 weeks, with increasing fibrotic changes on CT and incidences of exacerbation despite the reduction in TGF-β-mediated Smad activation.^78^^,^^79^ A dual αvβ6 and αvβ1 oral inhibitor (PLN-74809 on bexotegrast) is currently in phase 2 clinical trial.^80^ The completed phase 2a randomized, double-blinded, placebo controlled trial (INTEGRIS-IPF) has demonstrated the safety of PLN-74809 in IPF patients.^81^ Additionally, the exploratory endpoints showed that the average decline in FVC for patients on the placebo was 74.1 mL *vs.* 15.1 ml in the PLN-74809 group, with less fibrosis progression based on HRCT. The ongoing randomized, double-blind, placebo-controlled study (BEACON-IPF) evaluates the effect of 160 mg and 320 mg PLN-74809 on absolute FVC changes after 52 weeks of treatment with results expected as early as mid-2025.

***BI 1015550:*** BI 1015550 (nerandomilast) is a novel phosphodiesterase type 4 (PDE4) inhibitor that can abrogate fibroblast proliferation and differentiation into myofibroblast.^82^ BI 1015550 slowed the decline of FVC in patients with or without background anti-fibrotic treatment in a phase 2 double-blind, placebo-controlled trial over 12 weeks. In patients without pre-existing anti-fibrotic use, the median change in FVC was 5.7 mL in the BI 1015550 group and −81.7 mL in the placebo group. In patients with pre-existing anti-fibrotic use, the median change was 2.7 mL in the BI 1015550 group and −59.2 mL in the placebo group.^83^ The subsequent double-blind, randomized, placebo-controlled phase 3 trial evaluating two doses of BI 1015550 in improving lung function in IPF (FIBRONEER IPF) met its primary endpoint, the absolute change from baseline in FVC at week 52. This is the first IPF study which meets its primary endpoint in almost one decade.^84^

***Lysophosphatidic acid receptor-1 (LPA1) antagonist:*** LPA1 promotes fibrosis *via* mediation of the fibroblast recruitment in animal models.^85^ An LPA1 antagonist, BMS 986020, was evaluated in a phase 2, parallel-arm, randomized, double-blind, placebo-controlled trial over 26 weeks.^86^ A significant reduction in the rate of decline in the FVC at the end of 26 weeks was observed. Unfortunately, adverse reactions of hepatobiliary toxicity (elevated liver enzymes) and treatment-related cholecystitis led to the early termination of the study. A similar compound, BMS 986278 (admilparant), has been shown to be well tolerated and is able to slow the decline of lung function.^87^ At week 26, 60 mg twice daily admilparant reduced the rate of decline of predicted FVC by 1.4 % in IPF patients, compared to placebo. A new phase 3 study using BMS 986278 is recruiting and is expected to be completed in 2026.

***Phosphodiesterase 5 inhibitor:*** Treprostinil is an inhaled prostacyclin analog approved to treat pulmonary arterial hypertension. The INCREASE study investigated the efficacy of treprostinil in patients with pulmonary hypertension associated with ILD (PH-ILD). The primary endpoint is the change in peak 6-min walk distance from baseline at week 16. Patients using inhaled treprostinil had higher change from baseline in their 6-min walk test with the least-squares mean difference of 31.12 m.^88^ A *post hoc* analysis of the INCREASE trial found that inhaled treprostinil was associated with an improvement in the percentage of the predicted FVC of 1.8 %. Additionally, the beneficial effect of treprostinil is more pronounced in the IPF group compared with other PH-ILD groups, with a difference of 168.5 mL at week 16.^89^ Based on these data, two phase 3 randomized, double-blind, placebo-controlled studies (TETON and TETON-2) were initiated to evaluate the safety and efficacy of inhaled treprostinil in patients with IPF, with change in FVC as the primary outcome measure. A separate study focusing on treprostinil in patients with progressive pulmonary fibrosis (TETON-PPF) is also recruiting.

***N-acetylcysteine (NAC):*** IPF lungs are known to have increased levels of oxidative stress, and NAC is a known antioxidant.^90^^,^^91^ In 2005, a double-blind, randomized, placebo-controlled study assessed the effectiveness of NAC added to standard therapy with prednisone and azathioprine (Imuran) over 1 year. The authors concluded that adding NAC to the standard prednisolone and azathioprine (Imuran) therapy had better preserved vital capacity and DLCO (IFIGENIA).^92^ However, monotherapy NAC offered no significant benefits in preserving FVC in patients with mild to moderate lung function impairment compared to the placebo.^93^ Several randomized, placebo-controlled trials have examined the use of NAC monotherapy in treating IPF.^94^^,^^95^ The primary outcome was changes in FVC, and no significant difference was found between NAC and the placebo groups. Two randomized, placebo-controlled studies, including the PANORAMA study, examined NAC combined with pirfenidone in patients with IPF.^96^^,^^97^ Although neither found a significant difference in the incidence of adverse events, both studies found a greater decline in FVC in patients receiving NAC; however, both were limited by the small sample size. Recently, a *post hoc* analysis of the PANTHERIPF trial postulated that an individual's response to NAC may be predicted by their genetic mutation with favorability toward those with *MUC5B* mutations.^98^ This study also identified a subgroup of patients with the toll-interacting protein (*TOLLIP*) TT genotype in which NAC monotherapy was associated with a significant decrease in the composite endpoint of lung disease progression, hospital admission, transplantation, or death (hazard ratio 0.14). By contrast, the *TOLLIP* CC genotype was associated with a non-significant increase in the risk of the composite endpoint (hazard ratio 3.23). Based on these data, the phase 3 PRECISIONS trial is designed to compare the effect of NAC plus standard care in patients with IPF who have the *TOLLIP* TT genotype, highlighting a personalized medicine approach to treat IPF.

### Compounds failed in phase 2b/3 stage

***Ziritaxestat (GLPG1690):*** Autotaxin is elevated in many fibrotic conditions, and ziritaxestat is a potent and selective autotaxin inhibitor.^99^^,^^100^ The phase 2a randomized placebo-controlled trial (FLORA) found improvement in FVC in the treated patients (25 mL) *vs.* the placebo (−70 mL) at week 12.^100^ However, two large phase 3 trials, ISABELA 1 and 2, were unable to demonstrate any significant benefit of ziritaxestat when used alongside standard care treatments like pirfenidone or nintedanib. Both trials were terminated early due to the lack of efficacy in slowing the rate of FVC decline compared to placebo. In ISABELA 1, the FVC decline in the treatment groups did not show meaningful differences from the placebo group, and similar results were seen in ISABELA 2. Additionally, the trials raised concerns about higher all-cause mortality in the ziritaxestat-treated groups, particularly at the higher dose, which contributed to the decision to halt further development of the drug.

***Pamrevlumab (FG-3019):*** Pamrevlumab is a monoclonal antibody that binds to connective tissue growth factor (CTGF). The phase 2 PRAISE trial demonstrated that intravenous pamrevlumab reduced the decline in FVC by approximately 70 % in IPF patients at week 48 compared to the placebo group.^101^ However, the randomized, double-blind, placebo-controlled phase 3 study evaluating pamrevlumab in IPF patients failed to meet its primary endpoint (ZEPHYRUS-1). Despite pamrevlumab was found to be generally safe and well tolerated, a mean decline in FVC from baseline to week 48 of 260 mL in the pamrevlumab arm and 330 mL in the placebo arm at week 48 was detected. The trial also did not meet its secondary endpoint of time to disease progression, defined as an absolute percent predicted FVC decline of at least 10 % or death.^102^

***Zinpentraxin alfa (PRM-151):*** Pentraxin-2 (PTX2), also known as serum amyloid P, is a member of the pentraxin protein family and plays a critical role in fibrotic remodeling.^103^ Previous studies have demonstrated that circulating pentraxin levels are decreased in fibrotic disease.^104^ PTX2 inhibits macrophage pro-fibrotic polarization and TGF-β expression.^105^^,^^106^ A randomized, phase 2 double-blind study investigated if recombinant human PTX2 protein (PRM-151) infusion can reduce FVC decline in IPF subjects at week 28. In the PRM-151 treatment group, there was a reduction in decline in the percentage mean predicted FVC (−2.5 *vs.* −4.8) and this effect was sustained up to 52 weeks in patients enrolled in the open-label extension trial (predicted FVC decline of −3.6 %).^107^^,^^108^ Patients who crossed over to the PRM-151 treatment during the extension phase also had an improvement in percentage FVC decline from −8.7 % per year to −0.9 % per year. A phase 3 study evaluating the efficacy of PRM-151 (STARSCAPE) was discontinued in February 2023 due to futility, although the details were not disclosed.

***Olitigaltin (GB0139):*** GB0139 is a small molecule inhibitor of galectin-3 which is found to increase in the BAL fluid of patients with IPF.^109^^,^^110^ The phase 2b GALACTIC-1 trial evaluating the safety and efficacy of inhaled GB0139 did not meet its primary endpoint of change from baseline in the rate of decline in FVC and was discontinued. In the GALACTIC-1 trial, 173 patients who were not receiving pirfenidone or nintedanib, the current standard of care, received inhaled 3 mg dose of GB0139 or placebo over 52 weeks. The mean change in FVC from baseline to week 52 was −316.6 ml in the GB0139 3 mg group compared to −127.4 ml in the placebo arm. Higher incidences of adverse events were reported in 7.8 % of patients in the GB0139 group versus 1.4 % of patients in the placebo group.

### Novel agents in the phase 1/2a stage of clinical trials

***Senolytics (dasatinib plus quercetin):*** IPF is an aging-related disease, so it is tempting to target senescent cells as a therapeutic strategy. Dasatinib, a tyrosine kinase inhibitor, and quercetin, a flavonoid, were previously shown to be an effective senolytic agent *in vitro*.^111^ The first human open-label, pilot study examined this combination by administering intermittent dasatinib (100 mg/day) plus quercetin (1250 mg/day) orally over three consecutive days in three consecutive weeks in patients with IPF.^112^^,^^113^ Secondary endpoints were safety and change in functional and reported health measures. While there was significant improvement in the 6-min walk distance, there were no improvements in the FVC.^112^

***ABT-199 (venetoclax):*** Macrophages from IPF patients have increased expression of B-cell lymphoma-2 (Bcl-2) in mitochondria. In mice exposed to bleomycin, a conditional deletion of Bcl-2 in macrophages reversed established fibrosis by inducing macrophage apoptosis.^114^ Inhibiting Bcl-2 expression in macrophage mitochondria using Bcl-2 inhibitor, ABT-199 (Venetoclax), induced macrophage apoptosis. ABT-199 has shown therapeutic efficacy and good safety and tolerability in patients with chronic lymphocytic leukemia. In an ongoing phase 1 trial, investigators plan to evaluate the safety of venetoclax in IPF patients (NCT 05976217).

***Programmed death ligand 1 (PD-L1) antibody (atezolizumab):*** Immune checkpoint ligand PD-L1 has been found to increase in invasive lung fibroblasts and drives lung fibrosis in a humanized IPF model in mice. Activating PD-L1 in IPF fibroblasts promoted pulmonary fibrosis *in vivo*. PD-L1-neutralizing antibodies attenuated fibrosis *in vivo*, suggesting that PD-L1 may be a novel therapeutic target in IPF.^115^ In an ongoing phase 1 trial, investigators plan to evaluate the safety of atezoluzumab in IPF patients with or without standard of care therapy (NCT 05515627).

***Signal transducer and activator of transcription 3 (STAT3) inhibitor (TTI-101):*** The STAT3 protein is activated in lung fibroblasts and alveolar type II cells (ATII), thereby contributing to lung fibrosis in IPF.^116^ A phase 2 randomized, double-blind, placebo-controlled study plans to evaluate the safety and efficacy of TTI-101 in IPF patients with or without nintedanib (REVERT-IPF) (NCT 05671835).

In summary, despite significant advances in therapeutic development for IPF, several challenges remain. The shift from anti-inflammatory to anti-fibrotic treatments is exemplified by the failure of the PANTHER trial and the success of drugs like pirfenidone and nintedanib. However, these therapies only slow disease progression and do not halt or reverse the decline in lung function, emphasizing the need for more effective treatments. Current clinical trials exploring combination therapies, such as pirfenidone with nintedanib, have shown promising safety profiles, but efficacy data are limited, and no guidelines recommend combination therapy yet. Furthermore, several novel compounds, including integrin inhibitors, phosphodiesterase inhibitors, and autotaxin inhibitors, have entered clinical trials, but many have failed to meet primary endpoints or have been halted due to safety concerns. Future directions should focus on exploring new molecular targets, such as PD-L1 antibodies and senolytic therapies, which address underlying cellular mechanisms of fibrosis in addition to a background use of antifibrotics. Additionally, personalized medicine approaches, as seen with N-acetylcysteine and genetic markers like *TOLLIP*, hold promise but require further validation. The development of more robust biomarkers and early-stage compounds, combined with safer and more effective treatments, remains a critical goal in improving outcomes for IPF patients.

## Conclusions and future directions

Significant advances have been made in elucidating the biological processes that promote and sustain pulmonary fibrosis. Previously considered a chronic inflammatory disease mainly driven by dysregulated immune cells, IPF has now been viewed as a complex condition involving epithelial cells, immune cells, fibroblasts, and many others. Several novel therapies are in the pipeline that offers additional targets for treating IPF that could be used alone or as an add-on to nintedanib or pirfenidone. However, gaps in knowledge surrounding the treatment of IPF remain. The essential question is whether and how established fibrotic disease can be reversed and if normal lung tissue and function can be restored. There is also increasing interest and emphasis on regenerative medicine, mainly targeting epithelial and stem cells. The need for therapeutic trials based on specific biomarkers to develop personalized therapy for IPF patients continues to grow. Moreover, medications or formulations with better safety profiles are essential.

Nevertheless, early and accurate diagnosis of IPF is crucial given its high mortality rate at present, and minimally invasive approaches are under development. Incorporating clinical, radiographic, and molecular biomarkers, particularly harnessing the rapid technology development in AI, to accelerate early and accurate diagnosis remains a critical issue.

## CRediT authorship contribution statement

**Hongli Liu**: Writing–review & editing, Writing–original draft, Validation. **Jiaxi Shen**: Writing–review & editing, Writing–original draft, Visualization. **Chao He**: Writing–review & editing, Writing–original draft, Supervision, Project administration, Funding acquisition, Conceptualization.

## Funding

This work was supported in part by National Institutes of Health (NIH) grant K08HL163406.

## Declaration of competing interest

The authors declare that they have no known competing financial interests or personal relationships that could have appeared to influence the work reported in this paper.
